# The Effect of the Mediterranean Diet–Integrated Gamified Home-Based Cognitive-Nutritional (GAHOCON) Training Programme for Older People With Cognitive Frailty: Pilot Randomized Controlled Trial

**DOI:** 10.2196/60155

**Published:** 2024-12-13

**Authors:** Rick Yiu Cho Kwan, Queenie Pui Sze Law, Jenny Tsun Yee Tsang, Siu Hin Lam, Kam To Wang, Olive Shuk Kan Sin, Daphne Sze Ki Cheung

**Affiliations:** 1 School of Nursing Tung Wah College Hong Kong SAR China (Hong Kong); 2 School of Nursing and Health Sciences Hong Kong Metropolitan University Hong Kong SAR China (Hong Kong); 3 Mindvivid Hong Kong SAR China (Hong Kong); 4 Board of Director Office Pok Oi Hospital Hong Kong SAR China (Hong Kong); 5 School of Nursing and Midwifery Deakin University Burwood Melbourne Australia; 6 Alfred Health Victoria Australia; 7 School of Nursing The Hong Kong Polytechnic University Hong Kong China (Hong Kong)

**Keywords:** cognitive frailty, gamification, health education, Mediterranean diet, home based, cognitive training, older adults, geriatric, elderly, cognitive training, cognitive function, health education, intervention, nutritional education, cognitive impairment, dementia

## Abstract

**Background:**

Cognitive frailty is known to be associated with both nutrition and cognitive training. However, effective treatments that engage older adults with cognitive frailty in both the Mediterranean diet and cognitive training are lacking.

**Objective:**

This study aims to examine the feasibility and preliminary effects of Gamified Home-Based Cognitive-Nutritional (GAHOCON) on older adults with cognitive frailty, focusing on Mediterranean diet knowledge, adherence to the Mediterranean diet, cognitive function, physical frailty, grip strength, walking speed, memory, and body composition.

**Methods:**

This study applied a 2-center, assessor-blinded, 2-parallel-group, noninferiority, randomized controlled trial design. Eligible participants were community-dwelling adults aged 60 years or older, living with cognitive frailty, and exhibiting poor adherence to the Mediterranean diet. Participants were randomly assigned to the intervention or control group in a 1:1 ratio. In the intervention group, participants received 4 weeks of center-based training (health education) followed by 8 weeks of home-based training (GAHOCON). In the control group, participants received only the 4 weeks of center-based training and 8 weeks of self-revision of health educational materials at home. During the intervention period, time spent by the participants and the levels of difficulty completed by them weekly on GAHOCON were measured as markers of feasibility. The outcomes included Mediterranean diet knowledge, adherence to the Mediterranean diet, cognitive function, physical frailty, grip strength, walking speed, memory, and body composition. Data were collected at baseline (T0) and 1 week postintervention (T1). The Wilcoxon signed rank test was used to examine within-group effects for the outcome variables in each group separately.

**Results:**

A total of 25 participants were recruited, with 13 allocated to the intervention group and 12 to the control group. The median cumulative minutes spent on GAHOCON training increased from 117 to 926 minutes. The median level of difficulty completed for game 1 increased from level 14 to level 20, while for game 2, it increased from level 2 to level 24. After the completion of the interventions, Mediterranean diet knowledge was retained in the intervention group but significantly decreased in the control group (r=–0.606, *P*=.04). Significant improvements were observed in the intervention group in Mediterranean diet adherence (r=–0.728, *P*=.009), cognitive function (r=–0.752, *P*=.007), physical frailty (r=–0.668, *P*=.02), and walking speed (r=–0.587, *P*=.03), but no such improvements were seen in the control group.

**Conclusions:**

GAHOCON is feasible in engaging older adults with cognitive frailty to regularly participate in the intervention. Preliminary evidence suggests that it can retain Mediterranean diet knowledge following nutritional education, improve adherence to the Mediterranean diet, and enhance global cognitive function, physical frailty, and walking speed. However, the difficulty of the later levels of game 1 may be too high. Future studies should adjust the difficulty level of game 1. Additionally, trials with larger sample sizes and longer follow-up periods are needed to confirm its effects.

**Trial Registration:**

ClinicalTrials.gov NCT05207930; https://clinicaltrials.gov/ct2/show/NCT05207930

## Introduction

### Background

Cognitive frailty refers to the co-occurrence of physical frailty and cognitive impairment in the absence of dementia [1]. A systematic review reported a pooled prevalence of cognitive frailty of approximately 9%, with an increase from 6% during 2012-2017 to 11% during 2018-2020 [2]. Another systematic review found that cognitive frailty is a predictor of all-cause mortality and dementia [3]. People with cognitive frailty face a significantly higher risk of dementia and other adverse health outcomes (eg, malnutrition) compared with healthy individuals, as well as those with either mild cognitive impairment or physical frailty alone [4,5]. Cognitive frailty has been reported as a reversible condition, with greater potential for reversal at earlier stages [6].

Cognitive training refers to interventions designed to enhance domain-specific cognitive functions through repeated practice of theoretically driven skills and strategies [7]. Recently, cognitive training has transitioned from face-to-face formats to computerized platforms, offering improved cost-effectiveness, accessibility, and the ability to tailor content and difficulty levels to individual participants [8]. A systematic review reported that computerized cognitive training has positive effects on global cognitive function, memory, working memory, and executive function [9]. However, unlike cognitive stimulation—which engages individuals with cognitive deterioration in group activities and discussions to enhance cognitive and social functioning—or cognitive rehabilitation, which involves clients and their family members in achieving personally meaningful goals [10], most cognitive training content is not directly linked to daily living or overall quality of life [9]. For example, it often includes activities such as puzzle games [11]. Higher expectations of favorable effects are associated with increased odds of participating in computerized cognitive training [12]. Meaningful training content that is understandable to participants and relatable to their daily lives—for instance, tasks directly linked to everyday activities such as food selection—plays a crucial role in adherence to cognitive training. This is because participants recognize that the training can enhance their daily functioning, such as improving their ability to make food choices [13].

The Mediterranean diet is a dietary pattern typical of Greece and southern Italy in the 1960s. It emphasizes abundant plant foods and olive oil as the primary source of fat, with dairy products, fish, and poultry consumed in low to moderate amounts; red meat in low amounts; and wine in low to moderate quantities. Evidence in the literature indicates that greater adherence to the Mediterranean diet is associated with a lower risk of frailty in older adults [14-17], better global cognitive function, and a slower decline in global cognitive function among older adults without dementia [18]. The Mediterranean diet is not native to populations outside Mediterranean countries. Adherence to this diet may be influenced by factors such as cognitive function, sociocultural background, motivation, and lifestyle [19]. Evidence suggests that educational interventions promoting the Mediterranean diet can enhance cognitive health in cognitively healthy older adults [20]. However, the effectiveness of such interventions may be limited in populations unfamiliar with the Mediterranean diet and those with cognitive impairments.

Games engage and excite players, often fostering enjoyment and immersion—key characteristics of intrinsically motivated human behavior [21]. Gamification involves designing systems that replicate the experiences and motivations of games to influence users’ behaviors [22]. It is widely recognized as an effective approach to enhancing performance and motivation in educational settings [23]. Recent studies have shown that gamification has been successfully used and well accepted by older adults to improve their adherence to interventions (eg, cognitive training) and health behaviors (eg, physical activity) [24-26]. Therefore, gamification holds significant potential to engage older adults in sustained adherence to the Mediterranean diet through educational material revision and cognitive training. This study hypothesizes that gamification could engage older adults in participating in the Gamified Home-Based Cognitive-Nutritional (GAHOCON) intervention. The cognitive training component of GAHOCON may provide short-term improvements in cognitive function, while the dietary education component can help sustain knowledge of the Mediterranean diet through repeated revision of educational materials within the gaming process. As a result, health behaviors (eg, adherence to the Mediterranean diet) may be promoted, leading to improved clinical outcomes (eg, cognitive function, reduced frailty, physical performance). However, to date, no studies have examined the effects of a gamified program on promoting engagement with the revision of Mediterranean dietary educational materials and regular participation in meaningful cognitive training (ie, including dietary education) at home among older adults with cognitive frailty.

### Objectives

The objectives of this pilot trial were to examine the feasibility of the novel GAHOCON intervention, and compare the preliminary effects of the GAHOCON intervention with conventional health education on the following clinical outcomes in community-dwelling older adults with cognitive frailty: (1) retention of Mediterranean diet knowledge after health education, (2) adherence to the Mediterranean diet, (3) cognitive function, (4) physical frailty, (5) grip strength, (6) walking speed, (7) memory, and (8) body composition.

The selection of outcomes was based on evidence in the literature regarding the potential effects of dietary education on the Mediterranean diet (ie, Mediterranean diet knowledge [27], adherence to the Mediterranean diet [20], cognitive function [20], physical frailty [14,16], grip strength [28], walking speed [29], and body composition [28]) and cognitive training (ie, cognitive function [7,9,25], memory [9]).

## Methods

### Trial Design

This pilot study used a 2-center, assessor-blinded, 2-parallel-group, noninferiority, randomized controlled trial. The CONSORT (Consolidated Standards of Reporting Trials) 2010 guideline was followed to report on this trial [30]. This pilot trial was registered at ClinicalTrials.gov, with the last update on January 26, 2022 (ClinicalTrials.gov ID NCT05207930).

### Participants

Participants were recruited from 2 community centers for older adults, which provide various services (eg, health education, social and recreational activities) for community-dwelling individuals aged 60 years or older [31]. The research team created promotional materials, including flyers, and conducted recruitment talks at the centers to recruit participants. Staff members at the centers used the promotional flyers to invite interested and potentially eligible members to enroll in the study. Upon referral by the staff members of the centers, a research team member conducted eligibility screening at the centers. All eligible members were invited to participate in the study. The study’s inclusion criteria are presented in Textbox 1.

**Inclusion and exclusion criteria.**

**Inclusion criteria**

- Aged 60 years or older.
- Community-dwelling, as defined by living at home without residing in long-term care facilities (eg, nursing home) in the last 12 months.
- Cognitive frailty, as defined by the coexistence of mild cognitive impairment and physical frailty [32]:
  Mild cognitive impairment is defined by a Montreal Cognitive Assessment (MoCA) score of 25 or less and a Clinical Dementia Rating score of 0.5 [33,34].
  Physical frailty is defined by a Fried Frailty Phenotype (FFP) score of 1 or higher [35].
- Poor Mediterranean diet adherence, as defined by a Mediterranean Diet Score (MDS) of less than 30.5 (ie, the mean MDS of community-dwelling older adults reported in a local study) [16,36].
- Functionally independent in food preparation, as defined by a Lawton Instrumental Activities of Daily Living score of 15/18 or higher and a subscore for meal preparation of 2/2 [37].

**Exclusion criteria**

- Diagnosis of dementia, as defined by the documentation of the diagnosis in the participant’s medical records.
- Probable dementia, as defined by an MoCA score of 18 or less [33,38].
- Low vision (at least one eye with visual acuity <20/60 with corrective lenses, if available), as all the interventions are visual based.

### Interventions

#### Overview

Two interventions were implemented, which are detailed in the sections on the intervention group and control group. Apart from the interventions described below, the research team did not prohibit participants from engaging in any other activities during the intervention period. Participants were instructed to report any suspected adverse effects related to the intervention to the research team through the interventionists or the older adult center staff members as soon as they identified them. These events were recorded in a logbook maintained by a research team member (ie, RYCK).

#### Intervention Group

The intervention aimed to first provide health education to participants regarding cognitive frailty, cognitive training, and the Mediterranean diet. However, the knowledge gained from health education may not be easily remembered or internalized by older adults with cognitive frailty. Nonpurposeful cognitive training may also face poor adherence, as evidence shows that expectations toward participating in the study predict adherence to computer-based cognitive training [12]. By giving purpose to the cognitive training—through the revision of Mediterranean diet educational content—the intervention sought to enhance adherence to cognitive training, while also reinforcing participants’ knowledge of the Mediterranean diet to improve diet adherence.

As shown in Table 1, the intervention consisted of 2 components: (1) center-based training and (2) home-based training. The center-based training included a 2-hour lecture on the management of cognitive frailty, four 2-hour lectures (8 hours in total) on nutritional education regarding the Mediterranean diet, and two 1-hour sessions of GAHOCON technical training. The learning objectives of the health education were (1) understanding the concepts of cognitive frailty and its health implications, and (2) learning effective lifestyle modifications to mitigate cognitive frailty. The learning objectives of the nutritional education on the Mediterranean diet were 4-fold: (1) understanding the 11 types of food classifications according to the Mediterranean diet [39], (2) learning the 3-level classifications of food quality based on food preparation methods, (3) understanding the principles of portion exchange among foods within the same food category, and (4) learning the principles of food combination according to the Mediterranean diet. After completing the nutritional education, participants were provided with a handout summarizing the key points (Multimedia Appendix 1) from the four 2-hour lectures on the Mediterranean diet. They were instructed to refer to this handout as they practiced adhering to the Mediterranean diet. The learning objective of the GAHOCON technical training is to ensure participants can demonstrate the basic technical skills necessary to begin using GAHOCON at home. This includes turning on the tablet, connecting it to the internet, starting the game, and performing basic operations specific to the game (eg, object selection, object dragging). The center-based training was delivered during the first 4 weeks of the intervention, while the home-based training took place during the following 8 weeks. The entire intervention lasted for 12 weeks.

**Table 1:** Schedule of enrollment, intervention, and assessments.

Week								0				1				2				3				4				5				6				7				8				9				10				11				12				13
**Enrollment**																																																												
	Eligibility screen, informed consent, and group allocation						✓				N/A^a^				N/A				N/A				N/A				N/A				N/A				N/A				N/A				N/A				N/A				N/A				N/A				N/A	
**Interventions**																																																												
	**GAHOCON**																																																											
		**4-week center-based training (week 1-4)**				N/A				✓				N/A				N/A				N/A				N/A				N/A				N/A				N/A				N/A				N/A				N/A				N/A				N/A		
			Week 1: One 2-hour health education session		N/A				N/A				✓				✓				N/A				N/A				N/A				N/A				N/A				N/A				N/A				N/A				N/A				N/A			
			Week 2-3: Four 2-hour nutritional education sessions		N/A				N/A				N/A				N/A				✓				N/A				N/A				N/A				N/A				N/A				N/A				N/A				N/A				N/A			
			Week 4: two 1-hour GAHOCON technical training sessions		N/A				N/A				N/A				N/A				N/A				N/A				N/A				N/A				N/A				N/A				N/A				N/A				N/A				N/A			
		**8-week home-based training (week 5-12)**				N/A				N/A				N/A				N/A				N/A				✓				✓				✓				✓				✓				✓				✓				✓				N/A		
			**Dosage**																																																									
				30 minutes/day	N/A				N/A				N/A				N/A				N/A				N/A				N/A				N/A				N/A				N/A				N/A				N/A				N/A				N/A			
				5 days/week	N/A				N/A				N/A				N/A				N/A				N/A				N/A				N/A				N/A				N/A				N/A				N/A				N/A				N/A			
	**Control**						N/A				✓				N/A				N/A				N/A				N/A				N/A				N/A				N/A				N/A				N/A				N/A				N/A				N/A	
		**4-week center-based training (week 1-4)**				N/A				N/A				✓				✓				N/A				N/A				N/A				N/A				N/A				N/A				N/A				N/A				N/A				N/A		
			Week 1: One 2-hour health education session		N/A				N/A				N/A				N/A				N/A				N/A				N/A				N/A				N/A				N/A				N/A				N/A				N/A				N/A			
			Week 2-3: Four 2-hour nutritional education sessions		N/A				N/A				N/A				N/A				✓				✓				✓				✓				✓				✓				✓				✓				✓				N/A			
		**8-week home-based self-revision (week 5-12)**																																																										
**Data collection**																																																												
	Demographic						✓				N/A				N/A				N/A				N/A				N/A				N/A				N/A				N/A				N/A				N/A				N/A				N/A				N/A	
	Feasibility variables						N/A				✓				✓				✓				✓				✓				✓				✓				✓				✓				✓				✓				✓				N/A	
	**Outcome variables**						N/A				N/A				N/A				N/A				N/A				N/A				N/A				N/A				N/A				N/A				N/A				N/A				N/A				N/A	
		Mediterranean diet knowledge				N/A				N/A				N/A				N/A				N/A				N/A				N/A				N/A				N/A				N/A				N/A				N/A				N/A				N/A		
		Mediterranean diet adherence				N/A				N/A				N/A				N/A				✓				N/A				N/A				N/A				N/A				N/A				N/A				N/A				N/A				✓		
		Cognitive function				✓				N/A				N/A				N/A				N/A				N/A				N/A				N/A				N/A				N/A				N/A				N/A				N/A				✓		
		Grip strength				✓				N/A				N/A				N/A				N/A				N/A				N/A				N/A				N/A				N/A				N/A				N/A				N/A				✓		
		Waking speed				✓				N/A				N/A				N/A				N/A				N/A				N/A				N/A				N/A				N/A				N/A				N/A				N/A				✓		
		Memory				✓				N/A				N/A				N/A				N/A				N/A				N/A				N/A				N/A				N/A				N/A				N/A				N/A				✓		
		Verbal fluency				✓				N/A				N/A				N/A				N/A				N/A				N/A				N/A				N/A				N/A				N/A				N/A				N/A				✓		
		Body composition				✓				N/A				N/A				N/A				N/A				N/A				N/A				N/A				N/A				N/A				N/A				N/A				N/A				✓		

^a^N/A: not applicable.

**Figure 1:**
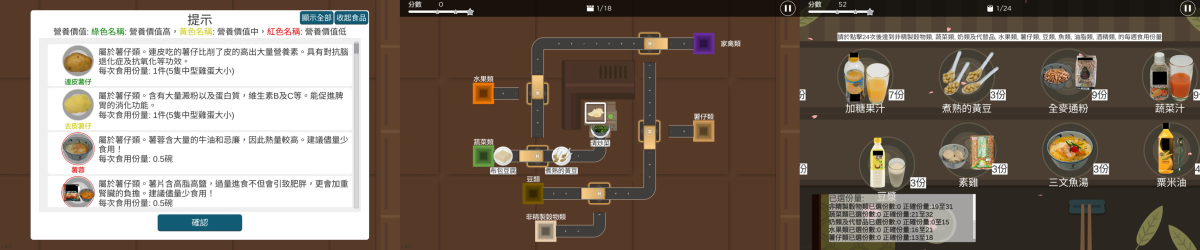
In-game sample pictures.

GAHOCON was codeveloped by a team of health care professionals, including nurse academics, clinical dieticians, social workers, and computer scientists from Mindvivid, a company specializing in developing computerized cognitive training for older adults. GAHOCON is compatible with tablets running either iOS (Apple Inc.) or Android (Alphabet Inc.). Two games, each focusing on different training objectives, were developed, with each game containing 30 levels of increasing difficulty (Multimedia Appendices 2 and 3). The difficulty level progressively increased, requiring participants to deepen their understanding of the Mediterranean diet and enhance their related cognitive functions. Between game levels, a revision break provided written and graphical materials summarizing the key points of the Mediterranean diet, aligned with the learning objectives of the nutritional education and relevant to the game content. Revising these materials helped participants build proficiency, enabling them to advance through the game levels more easily, as illustrated in Figure 1A. To ensure cultural relevance, the food images used in the GAHOCON game were created by the research team, featuring foods commonly available in local markets.

In game 1, as shown in Figure 1B, the game challenges participants’ attention and understanding of the classification of foods according to the Mediterranean diet (ie, learning objective 1 of the nutritional education on the Mediterranean diet). Food items, represented by pictures, enter from an opening and travel along a track. As they move, the items pass through switches that guide them onto different tracks. Participants are required to control the switches by tapping the relevant switch on the tablet to change the direction of the food items on the track. The goal of the game is to divert the food items to the ports corresponding to the correct Mediterranean food class. During the game, participants must focus their cognitive function—particularly attention—to match the food items on the track with the correct food class ports, which are labeled with the corresponding food class name, all within a limited time. If a food item on the track passes a port labeled with the corresponding name, it will be inserted into the wrong port, and the participant will not earn any points. As the game progresses, the difficulty increases: the speed of the food items on the track accelerates, the variety of food items expands, and the number of food ports available for participants to divert the food items into also increases. This progressively demands greater attention from participants in order to correctly move the food items to the right ports and score points.

In game 2, as shown in Figure 1C, the game challenges participants’ memory and understanding of the 3-level classifications of food quality according to food preparation methods (learning objective 2 of the Mediterranean diet nutrition education), the principles of portion exchange among foods within the same food type (learning objective 3), and the principles of food combination according to the Mediterranean diet (learning objective 4). In game 2, the food items (in the form of pictures) move along a reversible track. Participants are required to select a list of foods, considering the ideal amount, combination of food types, and food quality. During the game, participants need to engage their cognitive functions, particularly memory, to select the correct combination of foods according to the principles of the Mediterranean diet. This is because participants must remember their previous selections—if they choose repeated items (due to forgetting what they had already selected), those items will not be scored. Choosing repeated items prevents participants from advancing to the next game level. As the game progresses, the difficulty increases: the number of food items participants must select grows, and the variety of food items on the track expands. This increases the demand on memory function, as participants need to select the best combination of foods without repeating their choices to score points.

Evidence shows a positive dose-response relationship between cognitive training and cognitive improvement. Specifically, 5-6 days of training, lasting 25-30 minutes per day, has demonstrated significant cognitive improvement in older adults with mild cognitive impairment, with effects observable in as little as 2 weeks [40]. Additionally, a study indicated that an 8-week game-based Mediterranean diet learning program was effective in enhancing Mediterranean diet behaviors and global cognitive function [41]. To balance effectiveness and efficiency, participants were advised to engage in the GAHOCON intervention (either game 1 or 2) at home for 30 minutes per day, 5 days per week (ie, 150 minutes per week). The effects were measured after 8 weeks of training (ie, from week 5 to week 12). To ensure continuous participation, interventionists encouraged adherence to the recommended dosage by sending reminders, motivational messages, and emojis, and providing technical support to the participants. All tele-supervision measures were implemented via the digital communication platform WhatsApp (Meta Platforms, Inc.). The WhatsApp group consisted of 6-8 participants and was led by the interventionists.

During the intervention period, participants were provided with a tablet on loan to attend the GAHOCON training at home. To prevent cross-group contamination, GAHOCON was not publicly available for download, and each participant was assigned a unique log-in ID, ensuring that the training could only be accessed by participants in the intervention group. After completing the intervention, and at the participants’ request, GAHOCON was downloaded onto their smartphones, allowing them to continue the unfinished training at their discretion.

Although no adverse effects were reported in previous studies evaluating similar interventions (eg, computer-based cognitive training) [9,42,43], participants were instructed to report any adverse effects they suspected to be related to this study. Upon completion of the data collection at T1, participants in the intervention group were interviewed about any adverse effects related to GAHOCON experienced when returning their tablets to the research team. These reports were recorded in a logbook by the research team leader (RYCK).

#### Control Group

##### Control Group Training and Independent Learning Protocol

Participants in the control group attended the same center-based training, which included a 2-hour lecture on health education for managing cognitive frailty, and four 2-hour lectures (ie, 8 hours in total) on nutritional education about the Mediterranean diet, alongside participants in the intervention group during the first 3 weeks. Participants in the control group left the class in the 4th week, as they did not need to attend the GAHOCON technical training, as shown in Table 1. In the following 8 weeks, they were instructed to independently review the handout summarizing the key points of the Mediterranean diet covered in the four 2-hour lectures.

##### Outcomes

Data collection was conducted by trained research assistants, who demonstrated 100% interrater reliability. All procedures took place face-to-face at the older adult community center. Data were categorized into demographic and clinical variables, as well as outcome variables. Demographic and outcome variables were collected at baseline (T0). Mediterranean diet knowledge was not assessed at baseline, as the study aimed to examine knowledge retention following nutritional education. Therefore, the baseline (T0) assessment of Mediterranean diet knowledge was conducted after the nutritional education. All outcome variables were reassessed the week after the completion of the intervention (T1).

#### Demographic Variables

The following were considered: age, number of chronic illnesses as indicated by the Charlson Comorbidity Index [44], BMI, gender, education level, marital status, and living status.

#### Feasibility Variables

To examine the feasibility of participants navigating the progressively increasing difficulty levels of the game, the levels completed by participants (logged every Sunday during the intervention period) were recorded by the GAHOCON app. Both game 1 and game 2 consisted of 30 levels of difficulty.

To examine the feasibility of participants’ adherence to the GAHOCON intervention, participants were instructed to fill out an e-form sent via WhatsApp every Sunday, answering 2 questions: (1) “How many minutes, on average, did you play GAHOCON in the past 7 days?” and (2) “How many days did you play GAHOCON in the past 7 days?”

#### Outcome Variables

Mediterranean diet knowledge was assessed using a self-developed quiz, which consists of 20 multiple-choice questions designed to test participants’ understanding of the 4 learning objectives of nutritional education on the Mediterranean diet. The questions were created by an interprofessional team, including nursing academics and a dietitian. The quiz tested participants’ understanding of the 4 learning objectives covered in the 8-hour training on the Mediterranean diet. One point was awarded for each correct answer, with the total score ranging from 0 to 20. A higher score indicates a greater level of knowledge about the Mediterranean diet.

Mediterranean diet adherence was measured using the MDS [36], developed based on the method of Trichopoulou and colleagues [45]. The MDS includes 2 categories of items: beneficial foods (ie, nonrefined cereals, potatoes, fruits, vegetables, legumes, fish, and olive oil) and detrimental foods (ie, red meat and products, poultry, full-fat dairy products, and alcohol). Each MDS item is scored on a Likert scale from 0 to 5 to reflect the frequency of consumption. The total MDS is the sum of the 11 items, ranging from 0 to 55. A higher score indicates better adherence to the Mediterranean diet. The MDS has demonstrated good validity, showing a strong association with plasma and dietary fatty acids [39], as well as cardiovascular risks [36].

Cognitive function was measured using the MoCA. The MoCA consists of 30 dichotomous items, with 1 point awarded for each correct answer. The total MoCA score ranges from 0 to 30, with a higher score indicating better cognitive function. The MoCA has demonstrated a strong correlation with the Mini-Mental State Examination (*r*=0.90) and the Saint Louis Mental Status Examination (*r*=0.83) [46], as well as good criterion validity in detecting mild cognitive impairment [33].

Physical frailty was measured using the FFP [35]. The FFP quantifies frailty based on 5 components: weight loss, exhaustion, low physical activity, slow gait, and weakness. It has been validated to show good predictive validity for the incidence of major geriatric outcomes over 3-7 years, including falls, worsening mobility, hospitalizations, and death (hazard ratio 1.82-4.46) [35]. The FFP score ranges from 0 to 5, with 1 point assigned for the presence of each component. A higher FFP score indicates a greater level of physical frailty.

Grip strength was measured using the handgrip strength of the right hand, assessed with a Jamar dynamometer in kilograms. The Jamar dynamometer demonstrates good within-instrument reliability, with a high correlation of 0.82 for each instrument [47].

Walking speed was measured using the 6-minute walk test (6MWT). The 6MWT showed a strong correlation with the 10-meter walk test (*r*=0.94) and was also strongly correlated with other walking tests, including the 2-minute walk test, 6MWT, and 10-meter walk test (ρ=0.85-0.94). Additionally, it demonstrated excellent test-retest reliability (intraclass correlation coefficient 0.91-0.98) and interrater reliability (intraclass correlation coefficient 0.86-0.96) [48-50].

Memory was measured using the Fuld Object Memory Evaluation (FOME) [51]. The FOME requires participants to memorize and recall 10 objects 5 times, with a distraction task between each recall. The retrieval score is the sum of the recalled items across the 5 recall tests, ranging from 0 to 50 points. Participants are also asked to recall the 10 items again 20 minutes after completing the retrieval tests. The delayed recall score is the sum of the recalled items, ranging from 0 to 10, with a higher score indicating better memory. The distraction tests consist of 3 verbal fluency tasks, where participants are asked to recall names of animals, vegetables, and fruits within a specified time. The combined verbal fluency score is the sum of the items recalled across the 3 tasks, with a higher score reflecting better verbal fluency. The FOME has been validated in Hong Kong Chinese, demonstrating excellent test-retest reliability and good discriminative power in distinguishing dementia from normal cognitive function [52].

Body composition, including fat mass and skeletal muscle mass, was measured using the InBody 770 body composition analyzer (Biospace Inc.). This system uses an 8-point touch-sensitive electrode system, segmented bioelectrical impedance analysis, and multifrequency bioelectrical impedance analysis. The InBody 770 has demonstrated good reliability and agreement in measuring body composition when compared with dual-energy X-ray absorptiometry [53].

### Sample Size

No previous studies have reported the effects of an intervention like the one examined in this study. This pilot randomized controlled trial primarily aimed to estimate preliminary effects for the purpose of sample size estimation for a full-powered randomized controlled trial. Consequently, a precision sample size estimation method was not used. Literature suggests that a group size of 10-15 participants provides reasonable bias-corrected estimates for a pilot study aimed at estimating the effect size in a full-powered trial with medium to large effects [54]. Therefore, we aimed to recruit 25 participants, accounting for the 2 groups.

### Randomization

Permuted block randomization with block sizes of 8 and a 1:1 allocation ratio was used. A random allocation sequence was generated using the random function in Microsoft Excel, with each random number assigned a group label (ie, 1=intervention and 2=control). The generated numbers were then ranked from highest to lowest, and this process was repeated until the required number of participants (N=25) was reached for the random allocation sequence. An independent research assistant, who was not involved in any other aspect of the study, generated and maintained the random allocation sequence list. This assistant assigned group labels to participants based on the sequence of their entries, using the random allocation sequence list to ensure that the rest of the research team remained unaware of the group assignments.

### Blinding

Blinding of group labels was applied only to the outcome assessors. Outcome assessments were carried out by a team of trained research assistants who were blinded to the group labels and did not participate in any other part of the study. The group labels were not blinded for either the participants or the interventionists.

### Statistical Methods

Demographic and outcome data were described as medians with IQRs or frequencies with percentages and were compared between groups using the Mann-Whitney *U* test and Pearson chi-square test, depending on the level of measurement.

For objective 1, the weekly time spent on GAHOCON training during the home-based training period was described as the median cumulative minutes. The levels of difficulty achieved by the participants in the GAHOCON training each week during the home-based training period were also described by the median.

For objective 2, due to the small sample size, nonparametric tests were used to assess the preliminary effects. The Wilcoxon signed rank test was applied to examine within-group effects for continuous outcome variables in each group separately. Wilcoxon effect size *r* (*r*=*Z*/*N*^0.5^) was calculated to compare the effect size of each outcome between the 2 groups [55]. Missing data at T1 were handled using the last-value-carried-forward method. The level of significance was set at 0.05. An intention-to-treat analysis was conducted to interpret the hypothesis on the treatment effects [56].

### Ethical Considerations

The research protocol was reviewed and approved by the Institutional Review Board of The Hong Kong Polytechnic University (reference number HSEARS20211108005). Before data collection, all participants received detailed information about the study’s purpose, procedures, and their rights. Written informed consent was obtained from each participant, emphasizing their voluntary participation and the right to withdraw at any time without penalty. Participants’ identities were protected by assigning unique identifiers to each data set. Personal information was stored separately from research data and was accessible only to authorized research team members. Participation was completely voluntary, and participants were not compensated. The data collected were used solely for the purposes outlined in the study and were not shared with third parties. Results were reported in aggregate form to prevent the identification of individual participants.

## Results

### Participant Flow

**Figure 2:**
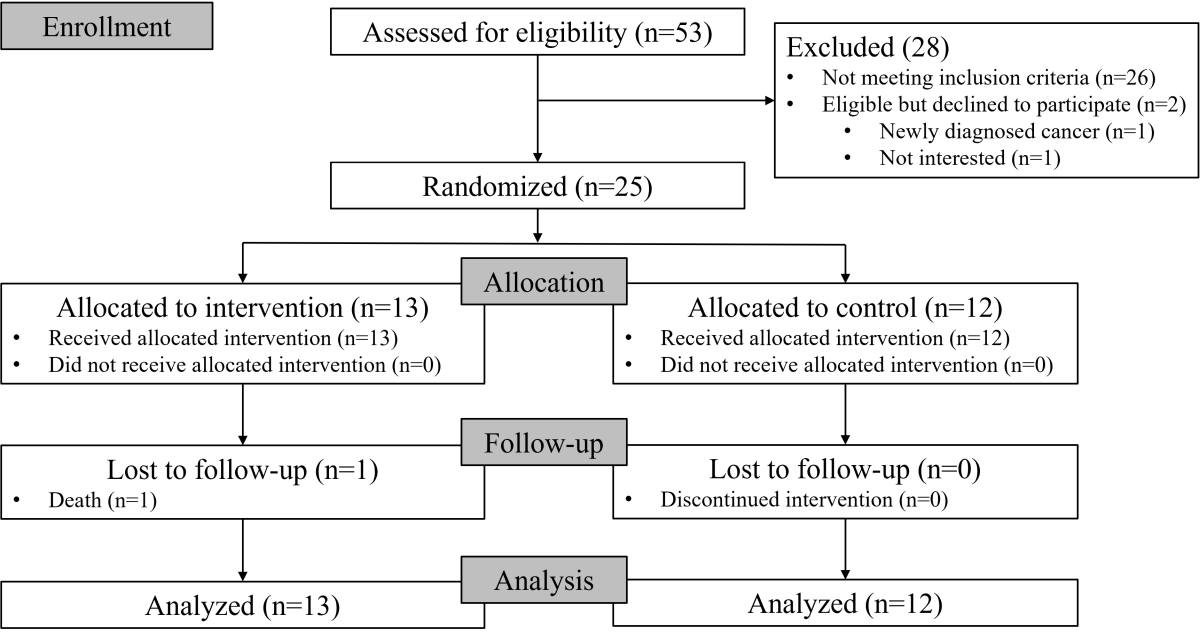
Participant flowchart​.

Participants were recruited from 2 community centers for older adults in Hong Kong. The study was conducted from February 1, 2023, to September 30, 2023. The trial concluded once the target number of participants was recruited, and the intervention and data collection were completed as planned. As shown in Figure 2, 53 older adults enrolled in the study and completed the eligibility assessment. Of these, 28 were excluded: 26 were ineligible, 1 chose not to participate due to a recent diagnosis, and 1 declined after gaining a better understanding of the study. A total of 25 participants joined the study (recruitment rate 47%) and were randomly allocated to either the intervention group (n=13) or the control group (n=12). All participants received their allocated interventions. However, 1 participant in the intervention group was lost to follow-up due to passing away during the intervention period. Additionally, data were missing for 4 participants in the control group regarding recall, delayed recall, and combined verbal fluency tests at baseline. The FOME tests for these 4 participants were not performed due to a shortened assessment time caused by unforeseen events at the older adult community center. The data from the participants who passed away at T1 were lost. Data from all remaining participants (n=25), including those with imputed missing values, were analyzed. See the completed CONSORT 2010 checklist presented as Multimedia Appendix 4 [30,57].

### Baseline Data

At baseline, as shown in Table 2, the participants had a median age of 70.0 years and a median BMI of 22.7 kg/m^2^. The majority were female (18/25, 72%), had no chronic illnesses (15/25, 60%), had attained secondary school education (13/25, 52%), were married (15/25, 60%), and were living with others (17/25, 68%). For the outcome variables, the participants’ median scores were as follows: Mediterranean diet knowledge, 15.0; MDS, 25.0; MoCA score, 23.0; FFP score, 2.0; grip strength, 15.5 kg; 6MWT completion time, 5.0 seconds; FOME-Retrieval score, 44.0; FOME-Delayed Recall score, 9.0; FOME Combined Verbal Fluency score, 34.0; fat mass, 17.4 kg; and skeletal muscle mass, 21.7 kg. There were no statistically significant differences between the intervention and control groups at baseline for any of these variables (Table 2).

**Table 2:** Demographic and outcome variables at baseline.

Variables			All (N=25)	Intervention (n=13)	Control (n=12)	*P* value
**Demographic variables**						
	Age (years), median (IQR)		70.0 (62.0-76.5)	74.0 (62.5-80.0)	68.5 (62.0-74.0)	.29
	**Number of chronic illnesses, n (%)**					.57
			0	15 (60)	8 (62)	7 (58.3)
			1-2	9 (36)	4 (31)	4 (33.3)
			>2	1 (4)	1 (8)	1 (8.3)
	BMI (kg/m^2^), median (IQR)		22.7 (20.2-25.8)	21.9 (20.5-25.4)	23.6 (20.1-27.5)	.69
	**Gender, n (%)**					.22
			Male	7 (28)	5 (38)	2 (16.7)
			Female	18 (72)	8 (62)	10 (83.3)
	**Education level, n (%)**					.29
			Tertiary	4 (16)	3 (23)	1 (8.3)
			Secondary	13 (52)	6 (46)	7 (58.3)
			Primary	6 (24)	2 (15)	4 (33.3)
			No education	2 (8)	2 (15)	0 (0)
	**Marital status, n (%)**					.38
			Unmarried	3 (12)	1 (8)	2 (16.7)
			Married	15 (60)	9 (69)	6 (50.0)
			Divorced	2 (8)	0 (0)	2 (16.7)
			Widowed	5 (20)	3 (23)	2 (16.7)
	**Living status, n (%)**					.26
			Live alone	8 (32)	2 (15)	6 (50.0)
			Live with others	17 (68)	11 (85)	6 (50.0)
**Outcome variables**						
	Mediterranean diet knowledge (Mediterranean diet quiz), median (IQR)		15.0 (12.0-16.0)	14.5 (12.0-16.8)	15.0 (11.0-16.0)	>.99
	Mediterranean Diet adherence (Mediterranean Diet Score), median (IQR)		25.0 (21.0-27.0)	26.0 (21.5-27.5)	24.0 (19.5-27.0)	.50
	Cognitive function (Montreal Cognitive Assessment), median (IQR)		23.0 (21.0-25.0)	24.0 (21.0-25.0)	23.0 (21.0-25.0)	.69
	Physical frailty (Fried Frailty Phenotype), median (IQR)		2.0 (1.0-3.0)	2.0 (2.0-3.5)	2.0 (1.0-2.0)	.06
	Grip strength (kg), median (IQR)		15.5 (13.0-18.0)	15.9 (14.5-18.0)	14.3 (8.9-18.6)	.41
	Walking speed (seconds; 6-meter walk test), median (IQR)		5.0 (4.0-6.8)	5.6 (4.1-7.3)	4.8 (4.0-5.8)	.44
	**Memory, median (IQR)**					
		Fuld Object Memory Evaluation-Retrieval	44.0 (38.5-45.0)	44.0 (31.5-46.0)	45.0 (41.3-45.0)	.65
		Fuld Object Memory Evaluation-Delayed Recall	9.0 (8.5-10.0)	10.0 (7.0-10.0)	9.0 (9.0-10.0)	.81
	**Verbal fluency, median (IQR)**					
		Fuld Object Memory Evaluation-Combined Verbal Fluency	34.0 (31.5-40.5)	35.0 (31.0-44.5)	32.0 (32.0-38.8)	.35
	**Body composition, median (IQR)**					
		Fat mass (kg)	17.4 (13.8-22.9)	16.2 (14.4-23.7)	17.7 (12.8-23.1)	.98
		Skeletal muscle mass (kg)	21.7 (18.3-24.7)	21.9 (18.1-24.7)	21.0 (18.7-25.3)	.98

### Feasibility Markers

**Figure 3:**
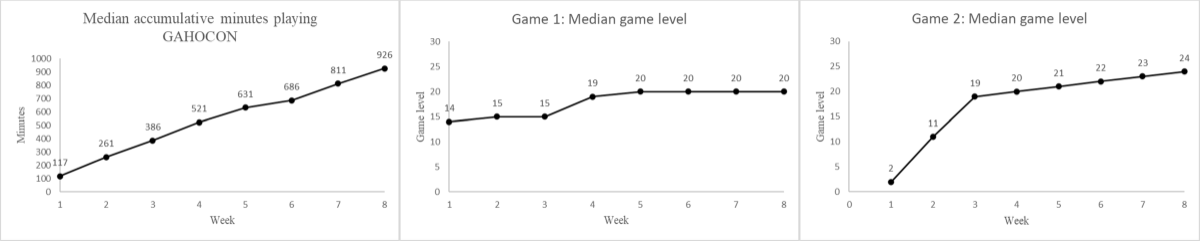
Engagement in the GAHOCON (Gamified Home-Based Cognitive-Nutritional) intervention.

As shown in Figure 3A, in the intervention group, the median cumulative minutes spent playing GAHOCON per participant increased from 117 minutes in week 1 to 926 minutes in week 8. As shown in Figure 3B, the median game level attained in game 1 rose from level 14 in week 1, peaked at level 20 in week 5, and remained stable through week 8. As shown in Figure 3C, the median game level attained in game 2 increased from level 2 in week 1 to level 24 in week 8, with no sign of leveling off.

### Preliminary Effects

As shown in Table 3, the median score of Mediterranean diet knowledge in the control group decreased significantly from 15.0 (IQR 11.0-16.0) at T0 to 12.5 (IQR 7.0-14.8) at T1 (*r*=–0.606, *P*=.04). By contrast, the change in the intervention group from T0 to T1 was not statistically significant, with a much smaller effect (*r*=–0.182, *P*=.51). The median MDS in the intervention group increased significantly from 26.0 (IQR 21.5-27.5) at T0 to 31.0 (IQR 27.3-34.0) at T1 (*r*=–0.728, *P*=.009), whereas the change in the control group from T0 to T1 was not statistically significant (*r*=–0.398, *P*=.17). Similarly, the median MoCA score in the intervention group increased significantly from 24.0 (IQR 21.0-25.0) at T0 to 25.0 (IQR 24.5-28.5) at T1 (*r*=–0.752, *P*=.007), while the change in the control group from T0 to T1 did not show statistical significance (*r*=–0.502, *P*=.08). The median FFP score in the intervention group decreased significantly from 2.0 (IQR 2.0-3.5) at T0 to 1.5 (IQR 0.5-2.0) at T1 (*r*=–0.668, *P*=.02), whereas the change in the control group from T0 to T1 did not show statistical significance (*r*=–0.567, *P*=.59). The median 6MWT completion time in the intervention group decreased significantly from 5.6 (IQR 4.1-7.3) at T0 to 4.5 (IQR 4.0-5.9) at T1 (*r*=–0.587, *P*=.03), while the change in the control group from T0 to T1 did not show statistical significance (*r*=–0.158, *P*=.58). There were no statistically significant changes in grip strength, memory (both FOME-Retrieval and FOME-Delayed Recall scores), verbal fluency, fat mass, or skeletal muscle mass in either the intervention group or the control group from T0 to T1. Throughout the study period, no untoward effects were reported by participants or older adult community center staff.

**Table 3:** Outcome estimates.

Outcomes and groups			T0, median (IQR)		T1, median (IQR)		*P* value		*r*
**Mediterranean diet knowledge (Mediterranean diet quiz)**									
	Intervention group	14.5 (12.0-16.8)		15.0 (11.0-17.5)		.51		–0.182	
	Control group	15.0 (11.0-16.0)		12.5 (7.0-14.8)		.04^a^		–0.606	
**Mediterranean diet (Mediterranean Diet Score)**									
	Intervention group	26.0 (21.5-27.5)		31.0 (27.3-34.0)		.009^a^		–0.728	
	Control group	24.0 (19.5-27.0)		29.0 (23.3-30.0)		.17		–0.398	
**Cognitive function (Montreal Cognitive Assessment)**									
	Intervention group	24.0 (21.0-25.0)		25.0 (24.5-28.5)		.007^a^		–0.752	
	Control group	23.0 (21.0-25.0)		24.0 (22.5-26.0)		.08		–0.502	
**Physical frailty (Fried Frailty Phenotype)**									
	Intervention group	2.0 (2.0-3.5)		1.5 (0.5-2.0)		.02^a^		–0.668	
	Control group	2.0 (1.0-2.0)		1.0 (0.3-2.8)		.59		–0.567	
**Grip strength (kg)**									
	Intervention group	15.9 (14.5-18.0)		19.2 (14.2-24.4)		.06		–0.514	
	Control group	14.3 (8.9-18.6)		17.8 (15.4-19.9)		.05		–0.567	
**Walking speed (seconds; 6-meter walk test)**									
	Intervention group	5.6 (4.1-7.3)		4.5 (4.0-5.9)		.03^a^		–0.587	
	Control group	4.8 (4.0-5.8)		4.8 (4.1-7.5)		.58		–0.158	
**Memory (Fuld Object Memory Evaluation-Retrieval)**									
	Intervention group	44.0 (31.5-46.0)		46.0 (38.5-47.0)		.21		–0.349	
	Control group	45.0 (41.3-45.0)		42.5 (34.3-45.0)		.26		–0.158	
**Memory (Fuld Object Memory Evaluation-Delayed recall)**									
	Intervention group	10.0 (7.0-10.0)		10.0 (9.3-10.0)		.17		–0.382	
	Control group	9.0 (9.0-10.0)		10.0 (8.0-10.0)		>.99		<0.001	
**Verbal fluency (Fuld Object Memory Evaluation-Combined verbal fluency)**									
	Intervention group	35.0 (31.0-44.5)		34.0 (28.0-40.5)		.81		–0.068	
	Control group	32.0 (32.0-38.8)		37.0 (31.5-38.8)		.13		–0.441	
**Body composition (kg; fat mass)**									
	Intervention group	16.2 (14.4-23.7)		18.0 (13.4-22.0)		.42		–0.223	
	Control group	17.7 (12.8-23.1)		18.1 (13.4-21.0)		.78		–0.079	
**Body composition (kg; skeletal muscle mass)**									
	Intervention group	21.9 (18.1-24.7)		22.6 (20.2-24.6)		.16		–0.388	
	Control group	21.0 (18.7-25.3)		20.6 (19.3-21.4)		.27		–0.317	

^a^*P*<.05.

### Harms

No adverse effects were reported by participants, either directly during the intervention period or in the postintervention interview regarding any potential adverse effects.

## Discussion

### Strengths

To the best of the authors’ knowledge, this is the first intervention of its kind, combining health education with a gamified training program that incorporates 2 therapeutic modalities (ie, Mediterranean diet education and cognitive training) for older adults with cognitive frailty to engage in at home.

### Principal Findings

The key findings are 3-fold. First, the GAHOCON intervention was feasible in engaging older adults with cognitive frailty, who participated continuously throughout the intervention period. They demonstrated steady progress in increasing the levels of difficulty, and the program was well accepted by the participants, with no cases of intentional dropout. Second, GAHOCON was more effective in retaining Mediterranean diet knowledge taught during the health education sessions, and it also resulted in improved adherence to the Mediterranean diet compared with providing written revision materials alone. Third, GAHOCON showed positive clinical outcomes, including improvements in cognitive function, physical frailty, and walking speed.

### Interpretation and Generalizability

Participants generally engaged regularly with GAHOCON throughout the 8-week intervention, with the time spent each week remaining consistent, even in the final week. This finding aligns with a systematic review suggesting that gamification positively influences adherence [58]. Moreover, this study is the first to demonstrate that gamification can sustain adherence to computerized cognitive training combined with Mediterranean diet education in older adults with cognitive frailty. Future studies should rigorously compare the adherence rates between gamified and nongamified cognitive training. Additionally, this study applied an arbitrary approach to gamified training, as the framework of gamification in eHealth and its impact on improving health outcomes remains poorly understood [59]. Further research is needed to identify effective gamification strategies for engaging older adults with cognitive frailty in training.

In game 1, most participants were unable to progress beyond level 20, which may suggest that the difficulty level after this point was too challenging for them. By contrast, in game 2, most participants continued to advance to higher levels, with no plateau in progress by week 8. This may indicate that the difficulty level of game 2 was more appropriate for the participants. In future studies, game 1 should be adjusted to better accommodate participants, particularly in terms of difficulty after level 20. To refine the difficulty level, further research should explore the factors that may limit participants’ progress, such as cognition-related challenges (eg, insufficient attention) or difficulties related to Mediterranean diet knowledge (eg, lack of familiarity with the Mediterranean diet).

GAHOCON preliminarily demonstrated effectiveness in retaining Mediterranean diet knowledge following health education. Previous studies have also shown that gamification positively impacts knowledge retention, particularly in the education of younger individuals [60]. Additionally, evidence supports that technology-enhanced educational tools, such as virtual reality, can improve patients’ satisfaction, knowledge, and understanding of patient education. Although other methods, such as the teach-back technique, have proven effective in enhancing knowledge retention among patients and leading to better health outcomes [61], teach-back may be challenging for older adults with cognitive impairment. By contrast, revising health knowledge through a technology-enhanced gamified program not only helped retain healthy diet knowledge but also provided enjoyment and excitement. Further studies should explore participants’ experiences with GAHOCON, a technology-assisted and gamified educational approach. Understanding their experiences will be crucial for further refining the intervention.

This study provides preliminary evidence that 8 weeks of GAHOCON training can lead to beneficial health outcomes. The retention of Mediterranean diet knowledge, coupled with a significant improvement in Mediterranean diet adherence, suggests that knowledge retention plays a key role in influencing health behaviors. It is well established that higher levels of health knowledge are positively associated with improved health behaviors [62]. However, providing booster educational training can be costly due to the involvement of human resources. This study showed that merely providing reading materials may not be effective in sustaining health knowledge 8 weeks after health education in older adults with cognitive frailty. By contrast, offering gamified, home-based, self-paced, and tele-supervised training accessible on a tablet could be an effective solution for sustaining both Mediterranean diet knowledge and adherence.

Apart from the improvement in adherence to the Mediterranean diet, favorable clinical outcomes were also observed, including improved global cognitive function, reduced frailty, and increased walking speed. However, no improvements were noted in body composition or domain-specific cognitive functions. These findings are consistent with previous research showing that computerized cognitive training effectively promotes cognitive functions in older adults with mild cognitive impairment [9]. However, most of the literature consists of observational studies demonstrating an association between adherence to the Mediterranean diet and reduced frailty [14,15]. While evidence from a randomized controlled trial suggests that Mediterranean diet interventions can improve cognitive function [20], randomized controlled trials examining the effects of such interventions on frailty remain scarce. This study is the first of its kind to provide preliminary evidence that a 12-week cognitive-nutritional intervention may reduce frailty. This proof-of-concept trial supports the potential of the Mediterranean diet–integrated GAHOCON training program to achieve favorable clinical outcomes, in addition to improving Mediterranean diet knowledge retention and adherence. Further studies with larger sample sizes and longer follow-up periods are needed to confirm the effects of GAHOCON on global cognitive function, frailty, and walking speed.

### Limitations

This study has several limitations. First, the small sample size limits the ability to confidently confirm the effects of the intervention. With the given sample size, interaction effects between time and intervention groups could not be determined. Second, there were missing baseline data for 4 control group participants on recall, delayed recall, and the combined verbal fluency test outcomes. The missing data for these variables in the control group may undermine confidence in the associated effects. Third, although the intervention group showed a statistically significant improvement in physical frailty, the effect size was comparable to that of the control group. Theoretically, increased adherence to the Mediterranean diet over a short period (ie, 12 weeks) may not be sufficient to improve physical frailty. This could be due to the potential effectiveness of other short-term lifestyle modifications, such as increased protein intake and physical activity (learned through health education), in improving frailty over similar durations (ie, 12-24 weeks) [63]. A longer follow-up period is needed to assess the potential added benefits of prolonged adherence to the Mediterranean diet compared with health education on other lifestyle modifications. Fourth, adherence to the Mediterranean diet was measured using a self-reported questionnaire, which may introduce bias. Although the same method was used for both the intervention and control groups, and a significant improvement was noted only in the intervention group, the improvement may have been influenced by a higher level of knowledge about the Mediterranean diet in the intervention group. This could have led participants to select answers that aligned with the expectations of the research team (ie, social desirability bias may have been more pronounced in the intervention group, as they were more likely to provide socially desirable responses in the self-reported questionnaire) [64], rather than reflecting actual adherence to a Mediterranean dietary pattern. The effect of the intervention in improving adherence to the Mediterranean diet should be interpreted with caution due to this limitation. Future studies may consider incorporating objective methods to measure Mediterranean diet adherence (eg, image-based dietary assessment methods) [65]. Finally, Mediterranean diet knowledge was not assessed at baseline, as the study aimed to examine knowledge retention following nutritional education.

### Conclusions

GAHOCON is feasible in engaging older people with cognitive frailty to attend the intervention regularly. Preliminary evidence also showed that it could retain Mediterranean diet knowledge after nutritional education; improve adherence to the Mediterranean diet; and improve global cognitive function, physical frailty, and walking speed. However, the difficulty of the later levels of game 1 might be too high. Further studies should adjust the level of difficulty of game 1. Trials with a large sample and a longer follow-up period are needed to confirm its effects.

## Appendix

Multimedia Appendix 1 Between-levels Mediterranean diet revision key points (texts are translated to English).

Multimedia Appendix 2 Game 1 (texts are translated to English).

Multimedia Appendix 3 Game 2 (texts are translated to English).

Multimedia Appendix 4 CONSORT (Consolidated Standards of Reporting Trials) checklist.

## Abbreviations

6MWT: 6-minute walk test
CONSORT: Consolidated Standards of Reporting Trials
FFP: Fried Frailty Phenotype
FOME: Fuld Object Memory Evaluation
GAHOCON: Gamified Home-Based Cognitive-Nutritional
MDS: Mediterranean Diet Score
MoCA: Montreal Cognitive Assessment

## Data Availability

The data sets generated during or analyzed during this study are available from the corresponding author upon reasonable request.
