# Supplementary figures and images for: The Effect of the Mediterranean Diet–Integrated Gamified Home-Based Cognitive-Nutritional (GAHOCON) Training Programme for Older People With Cognitive Frailty: Pilot Randomized Controlled Trial
Source: JMIR Rehabil Assist Technol. 2024 Dec 13;11:e60155. doi: 10.2196/60155 (PMC11681285; doi:10.2196/60155)

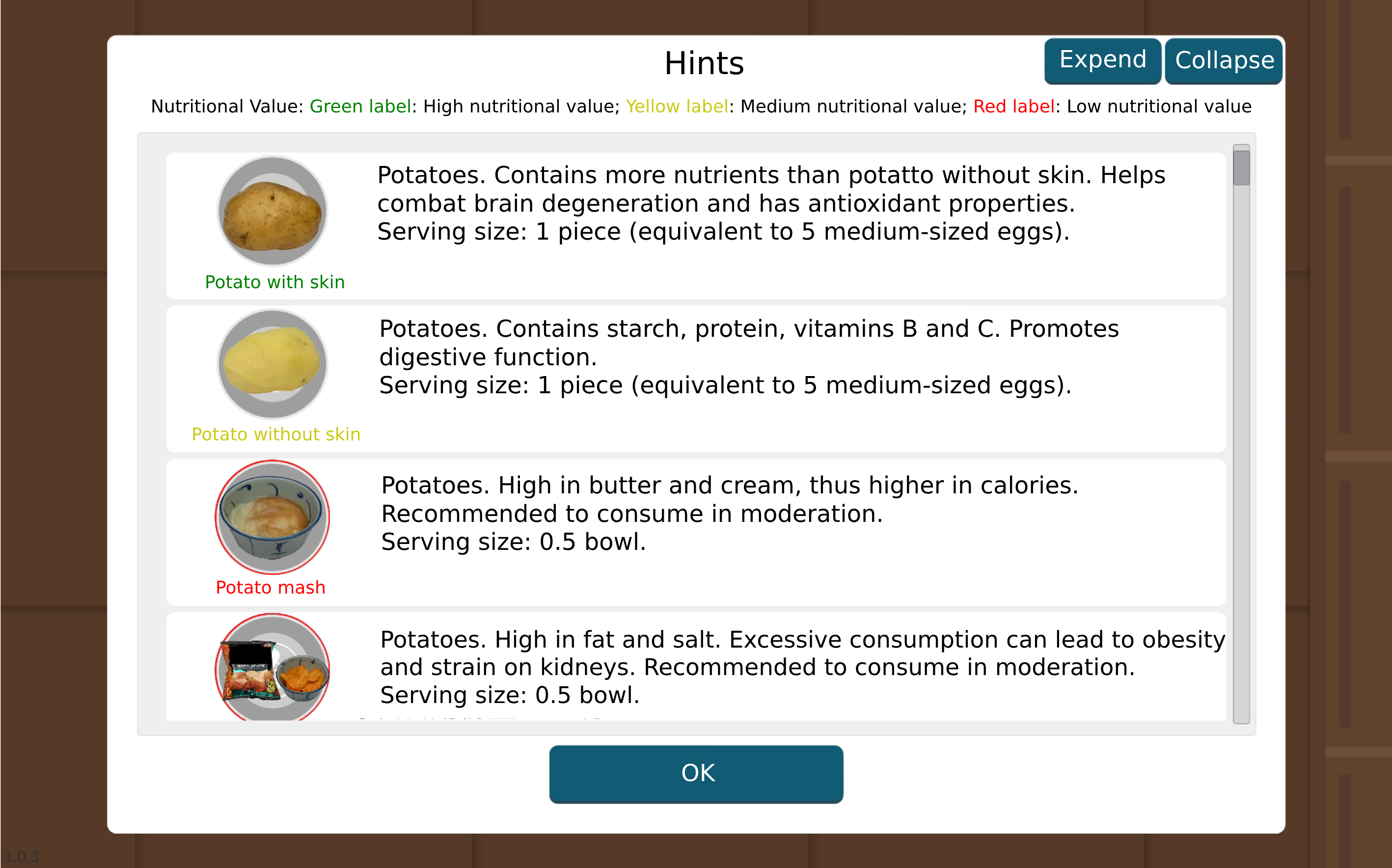

Supplement: Multimedia Appendix 1 [file rehab_v11i1e60155_app1.png]

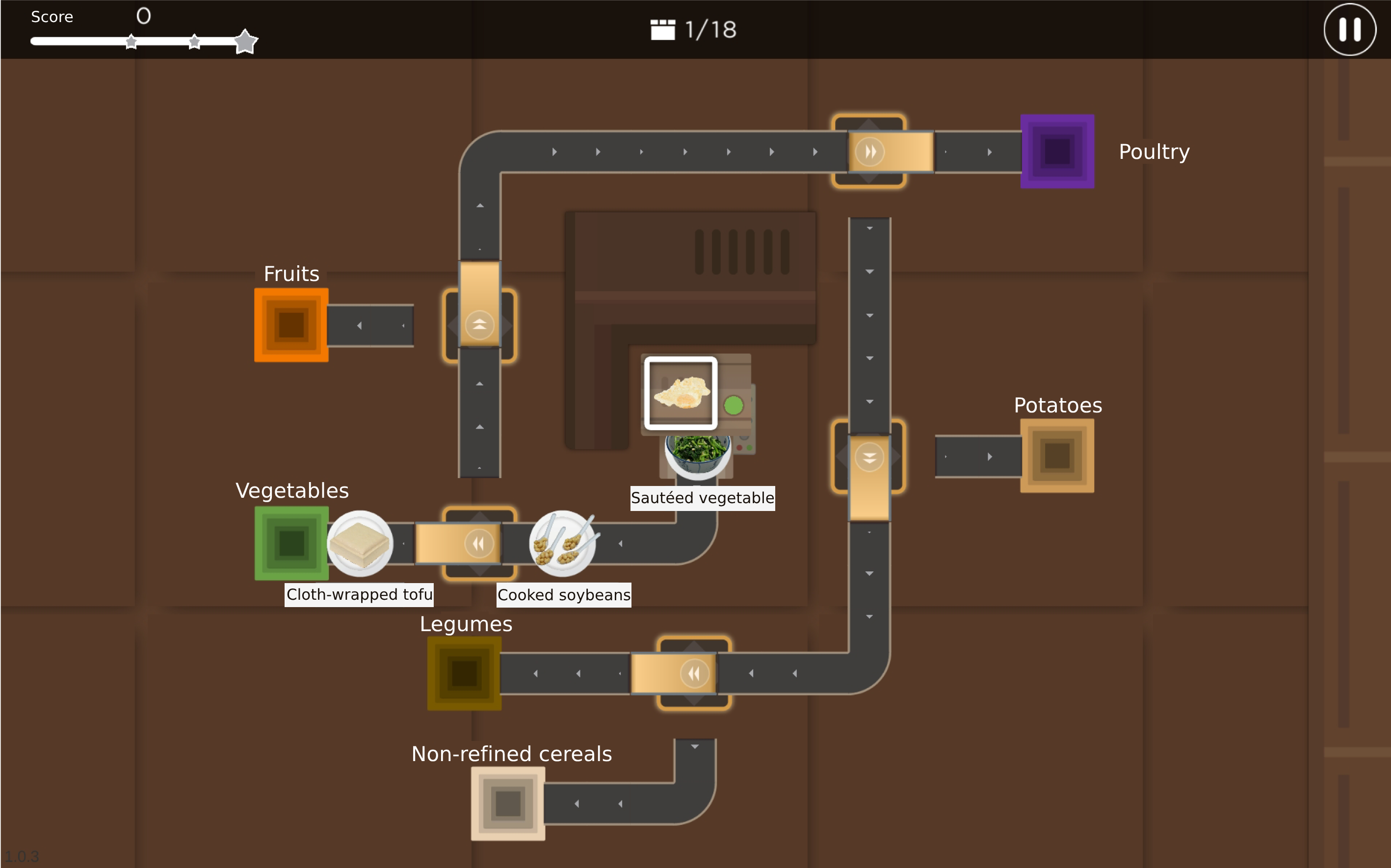

Supplement: Multimedia Appendix 2 [file rehab_v11i1e60155_app2.png]

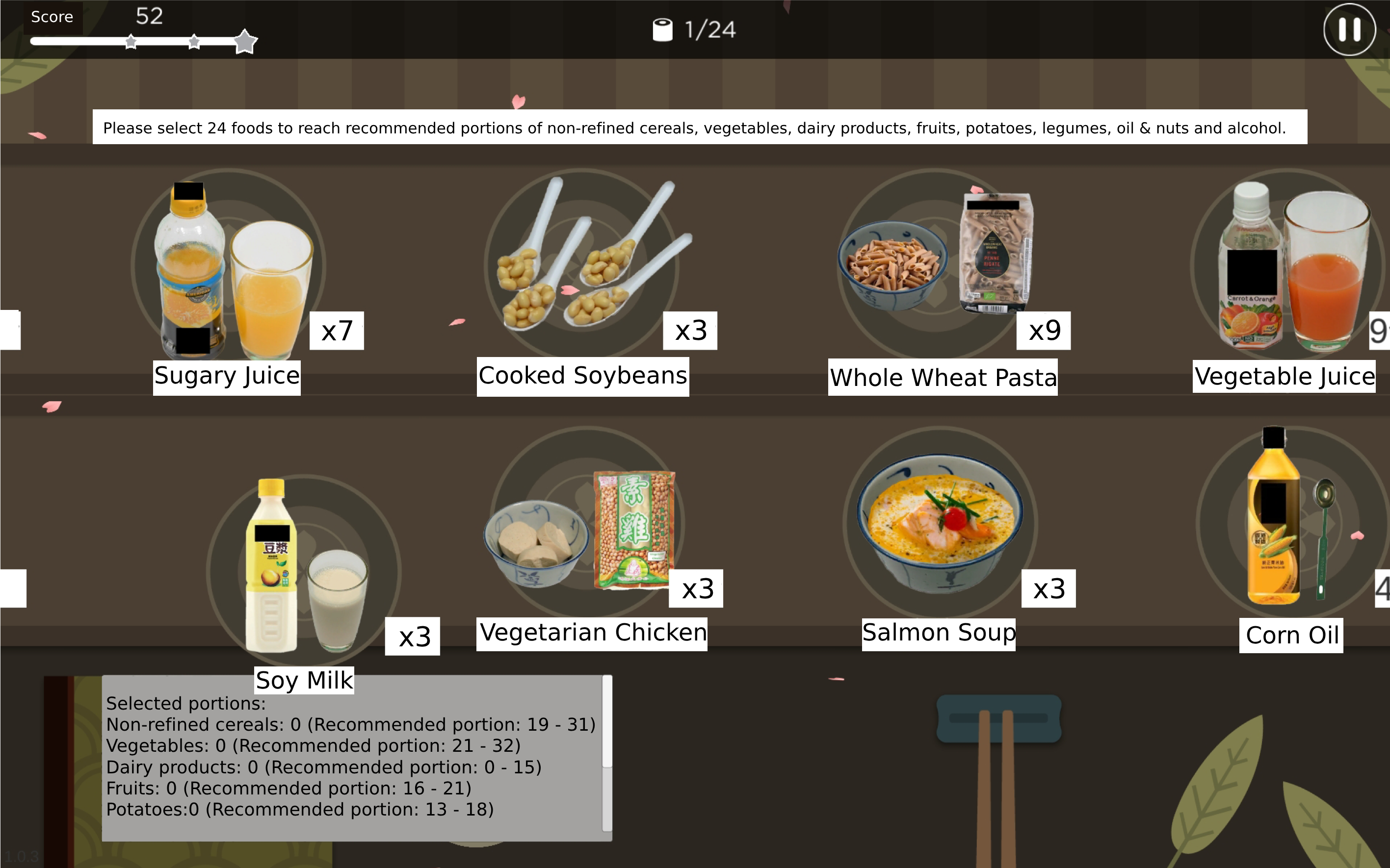

Supplement: Multimedia Appendix 3 [file rehab_v11i1e60155_app3.png]
