# Supplementary material for: The Effect of the Mediterranean Diet–Integrated Gamified Home-Based Cognitive-Nutritional (GAHOCON) Training Programme for Older People With Cognitive Frailty: Pilot Randomized Controlled Trial
Source: JMIR Rehabil Assist Technol. 2024 Dec 13;11:e60155. doi: 10.2196/60155 (PMC11681285; doi:10.2196/60155)
Supplement: Multimedia Appendix 4 [file rehab_v11i1e60155_app4.pdf]

CONSORT 2010 checklist of information to include when reporting a randomised trial*

Section/Topic	Item No	Checklist item	Reported on page No
**Title and abstract**			
	1a	Identification as a randomised trial in the title	Page2, Line41
	1b	Structured summary of trial design, methods, results, and conclusions (for specific guidance see CONSORT for abstracts)	Page2, Line45
**Introduction**			
Background and objectives	2a	Scientific background and explanation of rationale	Page3, Line85
	2b	Specific objectives or hypotheses	Page4, Line147
**Methods**			
Trial design	3a	Description of trial design (such as parallel, factorial) including allocation ratio	Page4, Line120
	3b	Important changes to methods after trial commencement (such as eligibility criteria), with reasons	Nil
Participants	4a	Eligibility criteria for participants	Page5, Line182
	4b	Settings and locations where the data were collected	Page5, Line174
Interventions	5	The interventions for each group with sufficient details to allow replication, including how and when they were actually administered	Page5, Line202
Outcomes	6a	Completely defined pre-specified primary and secondary outcome measures, including how and when they were assessed	Page10, Line336
	6b	Any changes to trial outcomes after the trial commenced, with reasons	Nil
Sample size	7a	How sample size was determined	Page11, Line413
	7b	When applicable, explanation of any interim analyses and stopping guidelines	Not applicable
**Randomisation**			
Sequence generation	8a	Method used to generate the random allocation sequence	Page12, Line423
	8b	Type of randomisation; details of any restriction (such as blocking and block size)	Page12, Line426
Allocation concealment mechanism	9	Mechanism used to implement the random allocation sequence (such as sequentially numbered containers), describing any steps taken to conceal the sequence until interventions were assigned	Page12, Line427
Implementation	10	Who generated the random allocation sequence, who enrolled participants, and who assigned participants to interventions	Page12, Line428
Blinding	11a	If done, who was blinded after assignment to interventions (for example, participants, care providers, those	Page12, Line433

		assessing outcomes) and how	
	11b	If relevant, description of the similarity of interventions	Not relevant because there is only one intervention
Statistical methods	12a	Statistical methods used to compare groups for primary and secondary outcomes	Page12, Line438
	12b	Methods for additional analyses, such as subgroup analyses and adjusted analyses	NA because there were no additional analysis
**Results**			
Participant flow (a diagram is strongly recommended)	13a	For each group, the numbers of participants who were randomly assigned, received intended treatment, and were analysed for the primary outcome	Page13, Line466
	13b	For each group, losses and exclusions after randomisation, together with reasons	Page13, Line475
Recruitment	14a	Dates defining the periods of recruitment and follow-up	Page13, Line468
	14b	Why the trial ended or was stopped	Page13, Line468
Baseline data	15	A table showing baseline demographic and clinical characteristics for each group	Page13, Line484
Numbers analysed	16	For each group, number of participants (denominator) included in each analysis and whether the analysis was by original assigned groups	Page15, Line498
Outcomes and estimation	17a	For each primary and secondary outcome, results for each group, and the estimated effect size and its precision (such as 95% confidence interval)	Page16, Line511
	17b	For binary outcomes, presentation of both absolute and relative effect sizes is recommended	NA, No binary outcome
Ancillary analyses	18	Results of any other analyses performed, including subgroup analyses and adjusted analyses, distinguishing pre-specified from exploratory	NA, No other analyses
Harms	19	All important harms or unintended effects in each group (for specific guidance see CONSORT for harms)	Page17, Line535
**Discussion**			
Limitations	20	Trial limitations, addressing sources of potential bias, imprecision, and, if relevant, multiplicity of analyses	Page19, Line613
Generalisability	21	Generalisability (external validity, applicability) of the trial findings	Page17, Line554
Interpretation	22	Interpretation consistent with results, balancing benefits and harms, and considering other relevant evidence	Page18, Line586
**Other information**			
Registration	23	Registration number and name of trial registry	Page1, Line39
Protocol	24	Where the full trial protocol can be accessed, if available	NA, no full trial

			protocol was published
Funding	25	Sources of funding and other support (such as supply of drugs), role of funders	Page20, Line653

Citation: Schulz KF, Altman DG, Moher D, for the CONSORT Group. CONSORT 2010 Statement: updated guidelines for reporting parallel group randomised trials. BMC Medicine. 2010;8:18.
© 2010 Schulz et al. This is an Open Access article distributed under the terms of the Creative Commons Attribution License (http://creativecommons.org/licenses/by/2.0), which permits unrestricted use, distribution, and reproduction in any medium, provided the original work is properly cited.

*We strongly recommend reading this statement in conjunction with the CONSORT 2010 Explanation and Elaboration for important clarifications on all the items. If relevant, we also recommend reading CONSORT extensions for cluster randomised trials, non-inferiority and equivalence trials, non-pharmacological treatments, herbal interventions, and pragmatic trials. Additional extensions are forthcoming: for those and for up-to-date references relevant to this checklist, see www.consort-statement.org.
